# Upper Airway Anatomical Landmark Dataset for Automated Bronchoscopy and Intubation

**DOI:** 10.1038/s41597-025-06169-0

**Published:** 2025-12-03

**Authors:** Ruoyi Hao, Yang Zhang, Zhiqing Tang, Yang Zhou, Lalithkumar Seenivasan, Catherine Po Ling Chan, Jason Ying Kuen Chan, Shuhui Xu, Neville Wei Yang Teo, Kaijun Tay, Vanessa Yee Jueen Tan, Jiun Fong Thong, Kimberley Liqin Kiong, Shaun Loh, Song Tar Toh, Chwee Ming Lim, Hongliang Ren

**Affiliations:** 1 Department of Electronic Engineering, The Chinese University of Hong Kong, Hong Kong SAR, China; 2 School of Mechanical Engineering, Hubei University of Technology, Wuhan, China; 3 School of Mechanical Science and Engineering, Huazhong University of Science and Technology, Wuhan, China; 4 Department of Biomedical Engineering, National University of Singapore, Singapore, Singapore; 5 Department of Otorhinolaryngology, Head and Neck Surgery, The Chinese University of Hong Kong, Hong Kong SAR, China; 6 Department of Otorhinolaryngology-Head and Neck Surgery, Singapore General Hospital, Singapore, Singapore; 7 Shun Hing Institute of Advanced Engineering, The Chinese University of Hong Kong, Hong Kong SAR, China

**Keywords:** Endoscopy, Biomedical engineering, Respiratory tract diseases

## Abstract

Bronchoscopy and intubation play crucial roles in respiratory disease diagnosis and treatment, yet the automation of their initial insertion phase remains limited. Advanced image analysis presents a viable solution to this challenge. However, insufficient comprehensive, publicly available datasets for training such models have hindered progress. We present a novel Upper Airway Anatomical Landmark (UAAL) Dataset, which annotates multiple anatomical landmark classes visualized through a bronchoscope, including the nose, nostril, channel, glottis, glottic aperture, vocal fold, and trachea, encompassing the entire upper respiratory tract from the nasal cavity to the trachea. It includes 3,814 clinical images from 82 patients with 10,330 annotations (4,910 instance segmentation masks and 5,420 bounding boxes) across 8 classes and 2,746 supplementary phantom images with 4,526 annotations (2,795 instance segmentation masks and 1,551 bounding boxes) across 9 classes. With its key contributions of diverse anatomical coverage, clinical data, supplementary phantom data, and public accessibility, this dataset will contribute to bronchoscopy and intubation automation systems, facilitating their transition from laboratory to clinical applications.

## Background & Summary

Respiratory diseases affect over 500 million people worldwide annually^1,2^, including both acute conditions like pneumonia and influenza, and chronic conditions such as asthma and chronic obstructive pulmonary disease (COPD). These diseases cause significant health and economic problems, requiring advanced diagnosis and treatment. Bronchoscopy and intubation play a pivotal role in this context^3,4^. Both procedures share an initial insertion step: the endoscopic navigation of the bronchoscope from the nose, through the upper airway, and into the trachea. Bronchoscopy allows for direct visualization of the airways, which is crucial for diagnosing conditions such as pulmonary inflammation, infections, and lung cancer^3,5^. Intubation, on the other hand, establishes an artificial airway and facilitates mechanical ventilation^6^. This technique is essential for maintaining respiratory function in patients with acute respiratory failure or severe pulmonary diseases, particularly in critical care settings and during surgical anesthesia^7,8^. However, bronchoscopy and intubation are technically demanding procedures that require a high level of expertise to perform safely and effectively while minimizing patient discomfort and risk of injury^9,10^. This complexity often leads to differences in care quality between experienced and junior doctors^11,12^.

To address these challenges, researchers have explored robotic systems^13^ to assist these procedures. For instance, an AI co-pilot bronchoscope robot^11^ has been proposed to assist with bronchoscopy. This human-robot collaborative approach enables doctors, including those with limited experience, to navigate the bronchoscope more effectively during lung examinations. However, such systems primarily focus on navigation within the tracheobronchial tree, neglecting the initial insertion phase from the external body to the trachea^11,14,15^. Similarly, different robotic assistance approaches have been explored for intubation procedures. Some robot-assisted systems mainly focus on mechanical structures, and their feasibility has been validated via teleoperation^16–18^. While others have explored integrating sensing technologies for automation^19,20^, though challenges in endoscopic navigation remain. Therefore, to improve bronchoscopy and bronchoscope-guided intubation automation, further research should focus on the initial insertion process.

Common image-based endoscopic navigation techniques include lumen centralization^21^, visual odometry^22^, and narrow-band illumination enhanced feature extraction^23^. Their integration is more advanced in colonoscopy automation, benefiting from the lower GI tract’s regular anatomy^24^. However, performance is limited in bronchoscopy automation due to the upper airway’s complex morphology^20,25^.

To overcome this, deep learning methods for anatomical feature detection have been explored^26^. These methods offer greater adaptability in complex anatomies than traditional techniques but require high-quality training datasets. However, current upper airway landmark datasets remain limited (Table 1). Laves *et al*.’s open-source vocal folds dataset^27^ provides detailed segmentation annotations for 7 classes, including 5 related to human anatomy. However, this dataset is small and lacks diversity beyond the laryngeal region. The REALITI dataset^28^ and Liu *et al*.^29^ annotated bounding boxes for orotracheal intubation scenarios, but used phantom models only, without clinical data, and are not public. The BAGLS dataset^30^ established a 1-class glottis aperture segmentation dataset but struggles with images containing closed vocal folds. Wang *et al*.^31,32^ annotated 4-class bounding boxes in a mixed simulated and phantom dataset, though it lacks clinical data and broader regional coverage. For nasotracheal intubation, intuNav^33^ annotated 4-class bounding box features (nose, throat, glottis, trachea), and Wei *et al*.^34^ provided glottis segmentation masks, but both remain unpublished. The lack of high-quality public datasets hinders robust AI development for automated bronchoscopy and intubation. Our dataset addresses this limitation by providing the detailed anatomical annotations necessary for visual servoing-based autonomous navigation^35,36^.

**Table 1:** Comparison of upper airway anatomical landmark datasets.

Dataset	Findings/Labels	Size	Year	Availability
Laves^27^	Clinical (5 classes)	536 images	2019	Open
REALITI^28^	Phantom (4 classes)	Unknown	2020	Limited
BAGLS^30^	Clinical (1 class)	59,250 images	2020	Open
Wang^31,32^	Virtual & Phantom (4 classes)	1,194 & 203 images	2023	Open
IntuNav^33^	Clinical (4 class)	3,615 images	2023	Limited
Wei^34^	Clinical (1 class)	492 images	2024	Limited
Liu^29^	Phantom (3 classes)	750 images	2024	Limited
Hackman^43^	Clinical (1 classes)	2,507 images	2024	Open
**Ours**	**Phantom (9 classes)**	**2,746 images**	**2024**	**Open**
	**Clinical (8 classes)**	**3,814 images**	**2024**	**Open**

In this work, we introduce a novel UAAL Dataset with the following features: 1. Diverse Anatomical Coverage: This dataset annotates a wide range of anatomical features from the external nasal cavity to the trachea. 2. Clinical Data: This dataset includes 3,814 images of clinical data collected during nasopharyngoscopy procedures, with 10,330 annotations comprising 4,910 instance segmentation masks and 5,420 bounding boxes across 8 classes. 3. Supplementary Phantom Data: To facilitate early-stage prototyping and testing in labs, the dataset also includes 2,746 images collected using a bronchoscope in a commercial airway phantom model, with 4,526 annotations including 2,795 instance segmentation masks and 1,551 bounding boxes across 9 classes. 4. Public Release: This public dataset allows the broader community to access and utilize it for developing bronchoscopy and intubation automation systems.

## Methods

**Fig. 1:**
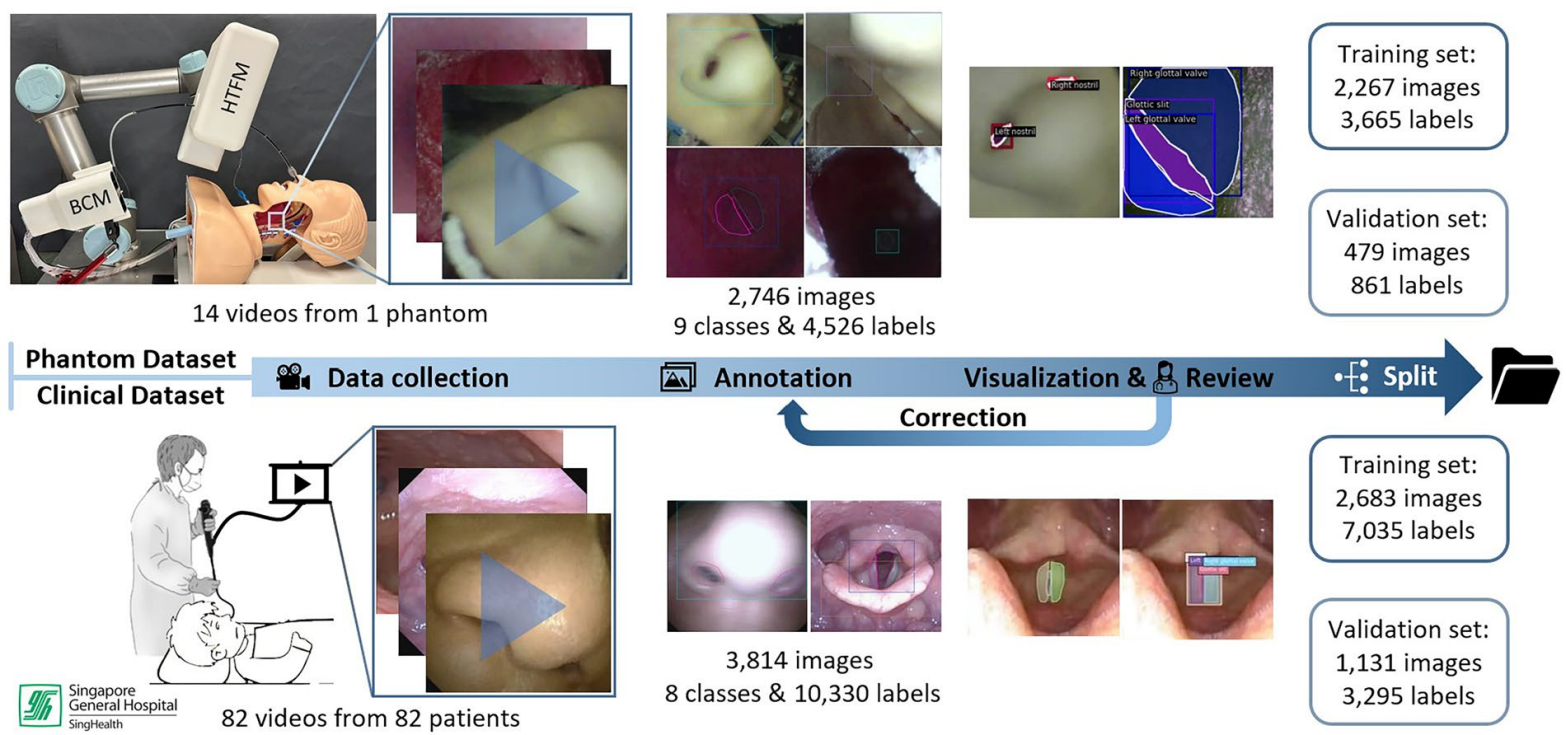
Workflow for constructing the UAAL dataset.

The UAAL Dataset annotates multiple anatomical landmark classes visualized through a bronchoscope, for bronchoscopy and intubation automation. It includes two subsets, created through a process shown in Fig. 1.

### Phantom data collection

The phantom dataset was collected in our research laboratory setting. Specifically, 14 videos were captured using an intubation robot system^35^ on a commercial airway phantom model. The intubation robot system, shown in the top-left of Fig. 1, consisted of the following components: 1) An experimental bronchoscope distal tip equipped with an embedded camera. The camera captures images at 400 × 400 pixels resolution and 30 frames per second (FPS). It uses an OV6946 (1/18-inch, CMOS) image sensor with a wide 120° ± 15% field-of-view, a 7 mm optimal working distance, and a depth of field ranging from 3 mm to 100 mm. Four integrated LED lights provided fixed, consistent illumination at the distal tip. The camera system utilized auto white balance and operated with a dynamic range of 68dB and a signal-to-noise ratio of 44dB, capable of functioning at a minimum illuminance of 0.03 Lux, ensuring clear visualization of the anatomical phantom under stable lighting conditions. 2) A bronchoscope control module (BCM) designed to control the bronchoscope distal tip articulation with high accuracy and responsiveness. 3) A holistic tube feeding module (HTFM) for smooth bronchoscope advancement and rotation. The commercial airway phantom model was purchased from the e-commerce platform Taobao. The brand is Taigui Medical, and the product is named “Human Tracheal Intubation Model” with the model number TG-J50. The phantom is made of PVC material and measures approximately 61 × 52 × 32 cm^3^ in size. This model is designed for training doctors in modern airway management techniques. It can be used to practice procedures such as orotracheal intubation, nasotracheal intubation, and nasopharyngoscopy. The left side of the head features a transparent wall, allowing for observation of the operational performance.

During data collection, the HTFM inserted the bronchoscope while the BCM simultaneously and dynamically adjusted its tip orientation. This coordination was crucial within the narrow channel region and around the anatomical structures of the glottis, which require precise navigation. This robotic control strategy allowed smooth guidance of the bronchoscope from the external nasal opening to the trachea, with the embedded camera recorded the whole process.

### Clinical data collection

The clinical dataset was obtained from clinical nasopharyngoscopy procedures conducted at the Singapore General Hospital. The source images of this dataset are from 82 videos, each obtained from a different patient. The patient cohort includes males and females aged 20 to 70 years old, providing a range of anatomical variations. These procedures were performed by experienced clinicians using commercial clinical flexible nasopharyngoscopes, representing real clinical situations, with over 95% of videos captured using two models: the Olympus ENF-VH (field of view: 110°, depth of field: 5.0-50 mm) and the Olympus ENF-V3 (field of view: 90°, depth of field: 3.50-50 mm). These scopes employ a fixed-focus lens system with automatically adjusted lighting, where the intensity of distal LED lights is dynamically optimized in real-time by the video processor based on reflected tissue light. The native image resolution was primarily recorded at native Olympus scope resolutions (e.g., 720 × 576 pixels), with some data sourced from surgical monitoring interfaces (e.g., 720 × 1280 and 1080 × 1920 pixels).

Although nasopharyngoscopy primarily focuses on the upper airway, the anatomical structures and landmarks visible during this procedure share significant similarities with those encountered during bronchoscopy and intubation. Both procedures involve navigation through the upper airway, including the nasal passages, pharynx, and larynx. Using nasopharyngoscopy data, we can create and test algorithms to identify key landmarks relevant to both bronchoscopy and intubation, linking these related procedures.

### Ethics declaration

The collection and publication of the clinical endoscopic image data in this study were conducted in strict compliance with ethical standards for human subject research. The IRB protocol, entitled “Endoscopic image recognition of nasopharyngeal cancer using deep learning network analysis,” was reviewed and approved by the SingHealth Centralised Institutional Review Board (CIRB) under the approval number 2020-3021. Prior to participation, all patients provided informed consent for the use and public sharing of their fully anonymized endoscopic images. The CIRB explicitly granted a waiver for the public dissemination of this de-identified data. All images utilized in the UAAL dataset were subjected to a rigorous de-identification process to remove all protected health information, ensuring complete patient anonymity and privacy.

### Annotation

The annotation process was carefully designed to ensure accuracy and consistency through a multi-stage, interdisciplinary approach. Three board-certified ENT specialists (each with >5 years of post-qualification experience from ENT specialization) first established the annotation guidelines through consensus. They created exemplary annotations, including 10 examples each for general structures (nose, nostrils, channel, trachea) and 50 examples each for finer structures (epiglottis, glottis, glottic aperture, vocal folds), setting the standard for subsequent labeling. A team of trained annotators (two PhD students and one professor specializing in medical AI and intelligent control) then performed the detailed labeling using the Computer Vision Annotation Tool (CVAT), a robust and user-friendly platform. To ensure initial quality control, each image was first annotated by one primary annotator, then underwent immediate cross-validation by the other two AI specialists. This internal review process resolved ambiguities and ensured technical consistency before specialist verification.

**Fig. 2:**
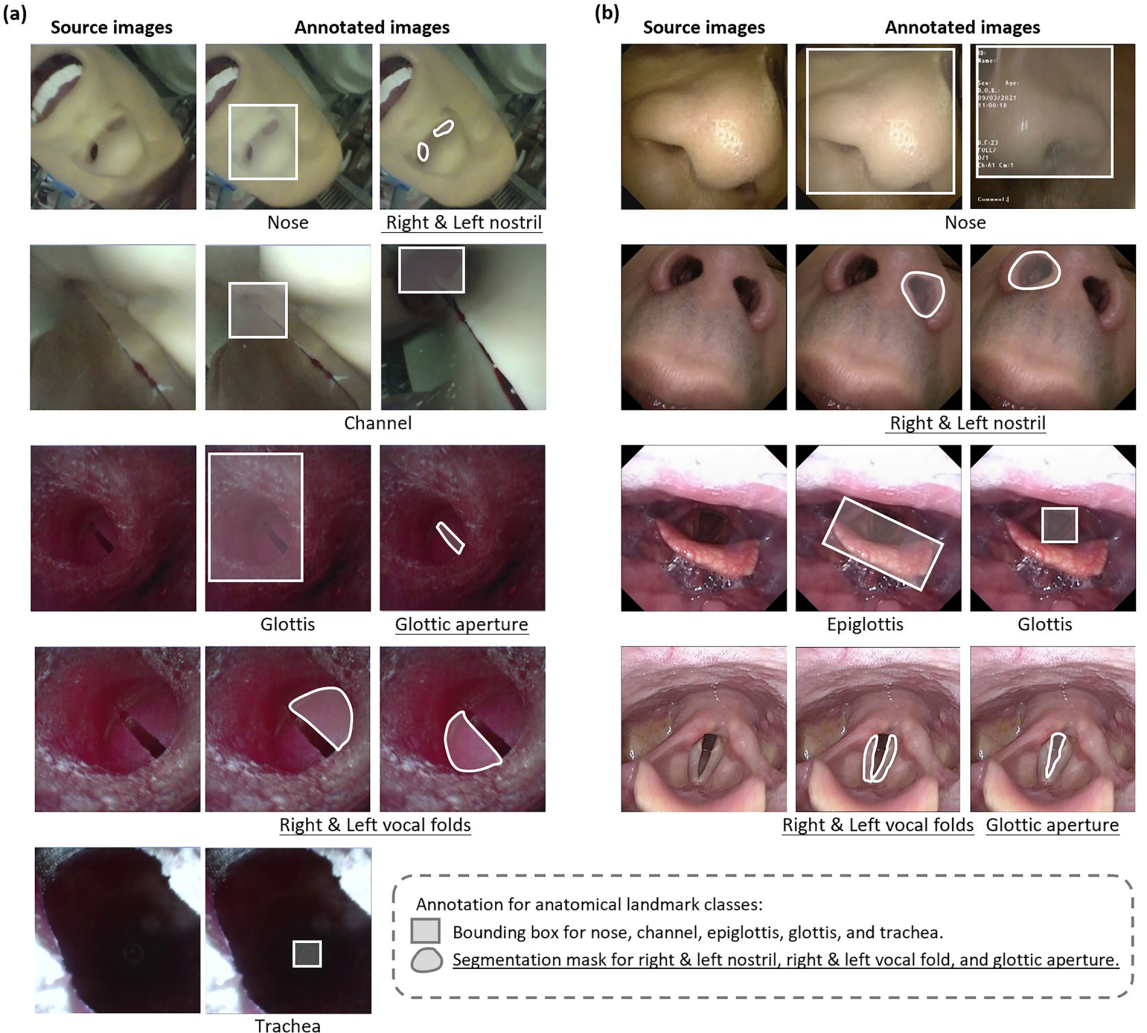
The typical samples of source and annotated images from phantom and clinical image datasets. (**a**) Phantom dataset. (**b**) Clinical dataset.

For the phantom dataset, as illustrated in the annotation example in Fig. 2(a), 9 distinct classes of anatomical landmarks were identified and annotated. We chose annotation strategies based on each landmark’s features. Bounding boxes were used to label more general structures such as the nose, channel, glottis, and trachea. This method efficiently captures the location and approximate size of these landmarks. Masks were employed to annotate structures with more clearly defined boundaries or more specific shapes, such as right nostril, left nostril, glottic aperture, right vocal fold, and left vocal fold, providing a pixel-level accurate representation of their shape and size. To ensure the highest quality for model training, a stringent manual quality control process was implemented; frames with significant motion blur were excluded to guarantee that all retained images feature clearly visible landmarks. Conversely, frames exhibiting minor, realistic optical artefacts such as vignetting (radial darkening at the periphery) and specular highlights (localized overexposure) were deliberately retained. These artefacts are representative of endoscopic imaging and do not obscure the core regions of interest. Their inclusion enhances the dataset’s utility for developing robust models capable of generalizing to real clinical environments.

Similarly, the clinical dataset, as shown in Fig. 2(b), comprises 8 annotated classes. Bounding boxes were used to mark the nose, epiglottis, and glottis, while masks were utilized to annotate structures right nostril, left nostril, glottic aperture, right vocal fold, and left vocal fold. Using both bounding boxes and masks helps represent each landmark clearly, which is useful for further analysis and model training. To ensure the clinical realism and practical utility of the dataset, our frame selection strategy encompassed variable image quality encountered in real-world procedures. As inherent characteristics of clinical endoscopy, various artifacts were present in the raw videos, including motion blur, temporary obstruction, and momentary focus variations. During frame extraction, we implemented a balanced quality control approach: while frames with extreme artifacts or complete obstructions were excluded to maintain diagnostic quality, those with mild to moderate motion blur, transient secretions, or other realistic, commonly encountered artifacts were retained through the standard selection process. Including such realistic variations is necessary for developing models that robustly generalize beyond idealized laboratory conditions to actual clinical environments.

### Visualization & review

After annotation, we exported the data from CVAT in the standardized Common Objects in Context (COCO)^37^ 1.0 format, which facilitated further data cleaning and validation. Subsequently, a comprehensive data cleaning process was implemented on the labeled data. This process involved removing incomplete or wrong annotations, standardizing label formats, and ensuring consistency in naming conventions across the dataset. Additionally, for all mask annotations, we generated corresponding bounding boxes representing the minimum enclosing rectangle (MER) for each mask. This step provided bounding box annotations derived from the mask annotations. The bounding boxes are useful for object detection tasks, while the combination of masks and bounding boxes enables instance segmentation tasks.

To ensure the quality and accuracy of the annotations, we utilized COCO visualization tools to create a comprehensive visual representation of our annotated data. This visualization included original images overlaid with both mask and bounding box annotations, color-coded representations of different anatomical landmark classes, and statistical summaries of annotation distributions across the dataset. The visualization tools allowed for both individual image inspection and batch review, enabling efficient identification of potential issues or inconsistencies.

Following this initial annotation phase, eleven independent ENT specialists (each with >5 years of post-qualification experience) carefully reviewed the visualization results. This review focused on the accuracy of anatomical landmark identification, precision of mask boundaries and bounding box placements, consistent labeling of similar structures, and proper representation of ambiguous cases. Any discrepancies or inaccuracies identified during this process were promptly documented and corrected. The corrections involved adjusting mask boundaries, refining bounding box positions and dimensions, correcting misclassified landmarks, and adding missing annotations or removing spurious ones. The goal was to establish a single, consensus ground truth. While all 11 specialists independently reviewed the data, their feedback was consolidated. Discrepancies or suggestions were not averaged but discussed and resolved to form a definitive corrected version. This iterative review and correction process continued until the supervising doctors, who provided expert medical oversight, confirmed that the dataset met the required standards of accuracy and consistency. The expertise of the two teams was strategically complementary. The ENT specialists provided authoritative domain knowledge to establish anatomical ground truth, while the AI specialists provided technical precision in label application and scalability. This interdisciplinary approach ensured both clinical relevance and computational usability of the resulting dataset. The internal cross-validation among AI specialists further enhanced technical consistency before medical verification.

### Split and packaging

The finalized annotations were split into training and validation sets following a meticulously designed methodology to ensure robust and unbiased model evaluation. The dataset was partitioned with three primary objectives. First, a conventional proportion allocation was followed, whereby approximately 70–80% of the images were assigned to the training set, with the remainder reserved for validation. Second, to prevent data leakage and avoid overly optimistic performance estimates, a video-level split was strictly enforced. This ensured that all frames originating from the same video sequence were contained entirely within either the training or the validation subset, thereby eliminating the risk of including highly correlated, nearly identical frames across both sets. Finally, a layered split was implemented to maintain a stratified distribution of annotation classes between the subsets. This approach guaranteed that each anatomical landmark was represented in both the training and validation sets, ensuring comprehensive evaluation across all classes and supporting the generalizability of the developed models. For the phantom dataset, the training set included 2,267 images with 3,665 labels, while the validation set contained 479 images with 861 labels. The clinical dataset training set had 2,683 images with 7,035 labels, and the validation set had 1,131 images with 3,295 labels. Please refer to the data analysis section for comprehensive details and distribution statistics.

## Data Records

**Fig. 3:**
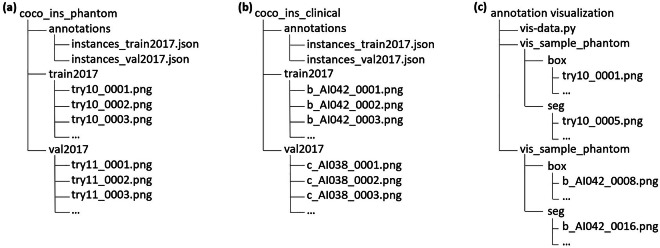
A visual overview of the dataset directory organization. (**a**) coco_ins_phantom.zip contains the phantom data. (**b**) coco_ins_clinical.zip contains the clinical data. (**c**) annotation visualization.zip provides supplementary files for result interpretation.

**Fig. 4:**
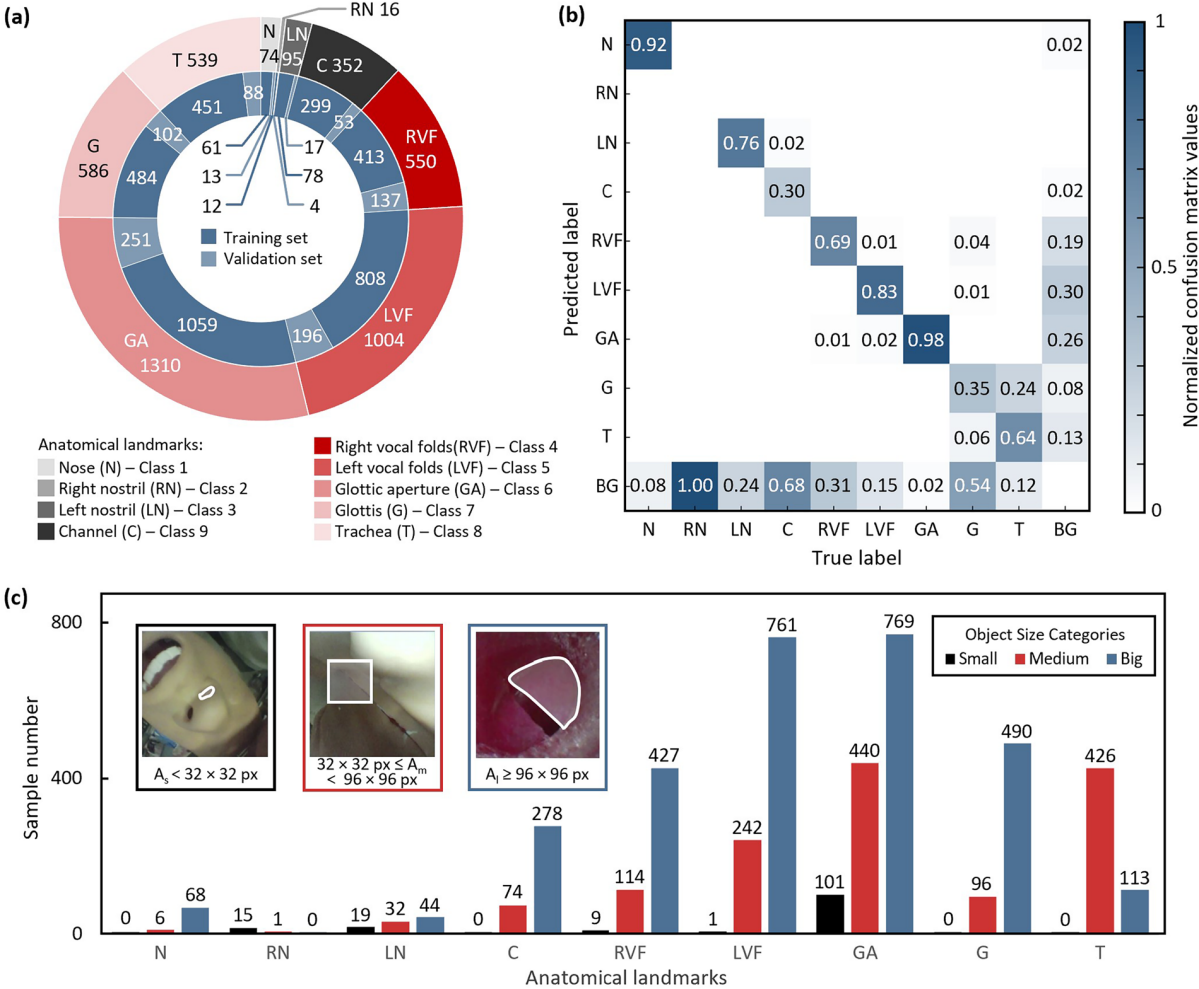
Statistics on classes in the phantom dataset. (**a**) Sample distribution. (**b**) Confusion matrices. (**c**) Annotated size-based counts.

**Fig. 5:**
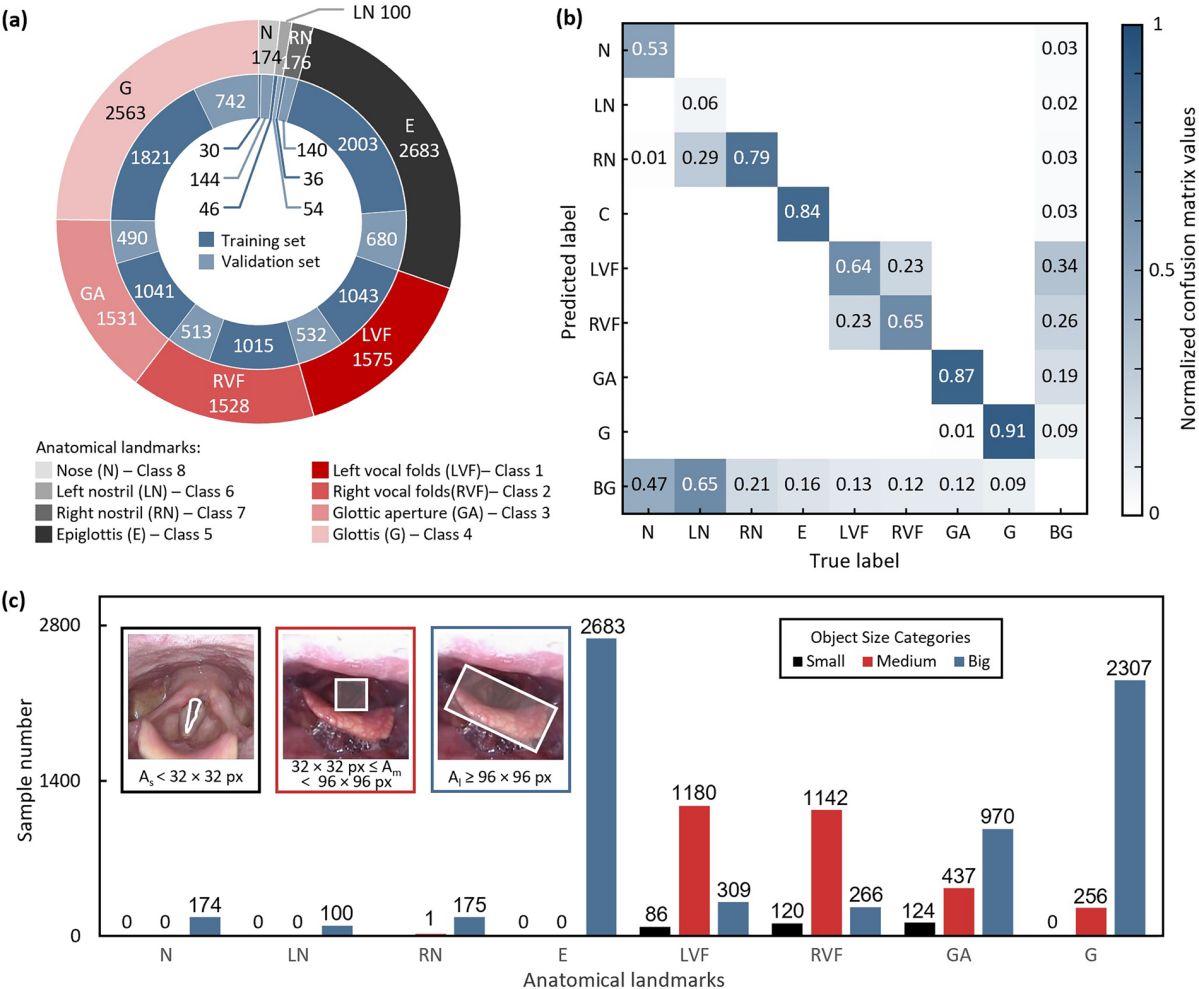
Statistics on classes in the clinical dataset. (**a**) Sample distribution. (**b**) Confusion matrices. (**c**) Annotated size-based counts.

The dataset is available on Figshare, an online open-access repository, at 10.6084/m9.figshare.26342779.v4^38^, with this section being the primary source of information on the availability and content of the data being described. The dataset is divided into three main components: the “coco ins phantom” for the UAAL-Phantom dataset, the “coco ins clinical” for the UAAL-Clinical dataset, and an “annotation visualization” sample. To provide a clear overview of the dataset’s organization, a directory tree structure is presented in Fig. 3, visually illustrating the contents of the publicly available ZIP files. Furthermore, a detailed summary of the directory structure and file contents is provided in Table 2. Each dataset contains these files: “train2017” and ‘val2017’ cover source images in the PNG format for the training set and validation set, and “annotation” covers two JSON-formatted annotation files for both the training and validation sets. All the labeled anatomical landmark classes are shown in Fig. 2, and the category IDs in the annotation file match the labels in Figure Legend below Figs. 4(a) and 5(a). For example, in the UAAL-Phantom dataset, Category 1 represents the nose (M), Category 2 represents the right nostril (RN), and Category 3 represents the left nostril (LN). While in the UAAL-Clinical dataset, Category 1 represents the right vocal fold (RVF), Category 2 represents the left vocal fold (LVF), and Category 3 represents the glottic aperture (GA). After specifying the class category IDs, the annotation file provides unique identification numbers for each source image. The last part contains detailed annotations. Specifically, the bounding box annotations for each annotated anatomical structure are stored in the “bbox” field, and segmentation mask annotations are stored in the “segmentation” field. Besides, for segmentation mask annotations, we also provide a second set of bounding box coordinates stored in the “bbox” field. These bounding box values represent the spatial extents that enclose the segmentation masks for each annotated anatomical structure.

**Table 2:** Summary of the dataset directory structure and contents.

Folder name	Subfolder name	File type	File count	Description
coco_ins_phantom	annotations	.json	2	Annotations
	train2017	.png	2267	Source images for training
	val2017	.png	479	Source images for validation
coco_ins_clinical	annotations	.json	2	Annotations
	train2017	.png	2683	Source images for training
	val2017	.png	1131	Source images for validation
annotation visualization	vis-data	.py	1	Visualization sample code
	vis_sample_phantom (box & seg)	.png	24	Phantom images visualization sample
	vis_sample_clinical (box & seg)	.png	17	Clinical images visualization sample

## Technical Validation

### Data analysis

Following the multi-stage quality control pipeline detailed in the Methods section, including frame screening, artifact management, and interdisciplinary annotation corrections, we obtained phantom and clinical datasets of high reliability and quality. Then, we analyzed both datasets in three ways. The sample distribution for each dataset is visualized in Figs. 4(a) and 5(a). The outer ring shows total annotations per category. The inner rings further break down the category counts between the training and validation sets. The results show the nose and nostril have a relatively small count. This is because patient facial images contain sensitive personal information, and images with large portions of the face are not suitable for public release. Additionally, external facial data is generally easier to obtain, so this dataset does not include a larger number of nostril annotations.

Then, to quantitatively assess the potential confusion between the different annotated categories, we performed a comprehensive evaluation of the model’s classification performance. The predictions were generated using deep learning models (a YOLO-based detector for bounding boxes and a YOLACT-based model for instance segmentation) applied to the validation set. Each prediction was required to meet dual criteria for correctness: precise localization, determined by an Intersection-over-Union (IoU) threshold of 0.5 against expert annotations, and accurate classification. The resulting comparisons were used to construct confusion matrices, which were normalized by the true labels (columns) to calculate recall and to clearly visualize error patterns independent of class imbalance. Figure 4(b) for the phantom dataset and Fig. 5(b) for the clinical dataset presents the confusion matrices computed on the training sets for both datasets. The results indicate that the left and right nostrils, as well as the left and right vocal folds, are the most commonly confused pairs of anatomical structures across both datasets. Other categories show relatively low cross-confusion. It is worth noting that researchers seeking to mitigate such issues of class imbalance and inter-class confusion in future work could consider strategies such as data augmentation (e.g., spatial and color transformations), specialized loss functions like focal loss, or network architectures designed for enhanced feature discrimination.

Lastly, we analyze the size distributions of the annotated anatomical structures. Following the widely adopted COCO object size standards, we categorized each annotation into three classes based on its pixel area: Small: area < 32 × 32 pixels; Medium: 32 × 32 pixels ≤ area < 96 × 96 pixels; Big: area ≥96 × 96 pixels. Figure 4(c) for the phantom dataset and Fig. 5(c) for the clinical dataset illustrate the distribution of annotation sizes across categories. The analysis reveals that the majority of annotations correspond to medium and large-sized anatomical structures. This size profile offers valuable insights into the composition of the dataset and may help guide the development of models robust to structural scale variations in real clinical settings.

### Benchmarking with SOTA models

To test our datasets, we selected several state-of-the-art (SOTA) methods in the field of object detection and segmentation for training and validation. Table 3 presents the detection and segmentation performance of SOTA methods on the phantom dataset. We evaluated model performance using common metrics such as *mAP*, *AP*_50_, and *AP*_75_. In terms of detection, RTMDet-Ins-s^39^ achieved the best *mAP*^*box*^ performance, reaching 40.6%. Compared to its performance on the COCO dataset, the accuracy dropped by approximately 4%. We attribute this performance drop primarily to the higher complexity of medical images, which are less compatible with general-purpose models, as well as the relatively limited dataset size, which may hinder some models from fully converging to their optimal performance. For segmentation, RTMDet-Ins-s achieved an *mAP*^*mask*^ of 39.7%, which is 1% higher than its accuracy on the COCO dataset.

**Table 3:** Comparison of accuracy with the state-of-the-art methods on the phantom dataset.

Methods	Backbone	Detection accuracy (%)			Segmentation accuracy (%)		
		*mAP*^*box*^	${\boldsymbol{A}}{{\boldsymbol{P}}}_{{\bf{50}}}^{{\boldsymbol{b}}{\boldsymbol{o}}{\boldsymbol{x}}}$	${\boldsymbol{A}}{{\boldsymbol{P}}}_{{\bf{75}}}^{{\boldsymbol{b}}{\boldsymbol{o}}{\boldsymbol{x}}}$	*mAP*^*mask*^	${\boldsymbol{A}}{{\boldsymbol{P}}}_{{\bf{50}}}^{{\boldsymbol{b}}{\boldsymbol{o}}{\boldsymbol{x}}}$	${\boldsymbol{A}}{{\boldsymbol{P}}}_{{\bf{75}}}^{{\boldsymbol{b}}{\boldsymbol{o}}{\boldsymbol{x}}}$
ATSS^44^	ResNet50	30.3	59.5	28.4	–	–	–
BoxInst^45^	ResNet50	33.2	54.5	36.4	13.4	36.5	11.1
CondInst^46^	ResNet50	33.5	49.7	35.8	30.0	46.5	34.0
CMask RCNN^47^	ResNet50	40.2	64.0	44.2	36.5	62.0	38.9
Conditional DETR^48^	ResNet50	15.6	37.3	10.2	–	–	–
DAB DETR^49^	ResNet50	18.4	44.2	13.9	–	–	–
Deform. DETR^50^	ResNet50	25.7	52.0	21.7	–	–	–
DETR^51^	ResNet50	6.7	17.3	5.2	–	–	–
GFL^40^	ResNet101	33.1	61.3	32.7	–	–	–
Mask2Former^52^	ResNet50	36.4	51.9	41.5	33.3	51.0	37.9
Mask RCNN^53^	ResNet50	33.3	54.9	32.6	33.5	54.7	34.3
Mask RCNN^53^	Swin-T	27.4	54.5	23.8	29.8	53.1	31.1
MS RCNN^54^	ResNet50	36.7	58.4	38.5	34.9	57.2	37.9
PointRend^41^	ResNet50	32.3	60.1	29.6	35.6	63.4	36.3
QueryInst^55^	ResNet50	9.8	19.8	8.0	11.0	21.3	11.0
SOLOv2^56^	ResNet50	–	–	–	36.2	57.3	42.0
SparseInst^57^	ResNet50	–	–	–	30.8	46.1	35.2
RTMDet-Ins-s^39^	CSPNeXt	40.6	63.7	46.5	39.7	61.9	46.5
YOLACT^58^	ResNet50	40.0	70.8	44.6	40.1	68.9	40.1

When comparing other SOTA models, the accuracy of each model showed slight fluctuations compared to their published accuracy on the COCO dataset, but there was no significant data shift. This fluctuation is normal because the characteristics of the features in our phantom dataset differ from those in the COCO dataset, causing general SOTA models to experience accuracy variations without fine-tuning. The results demonstrate that SOTA models can achieve similar performance on our phantom dataset as they do on the COCO dataset, indicating the high quality and validity of our phantom dataset.

For the clinical dataset, in Table 4, GFL^40^ achieved the best *mAP*^*box*^ performance, while PointRend^41^ achieved the best *mAP*^*mask*^ performance. Due to the more complex data features in the clinical dataset compared to the phantom dataset, and greater variations in the detection environment, all models experienced a loss in accuracy. Overall, the clinical dataset is a more challenging dataset that is closer to real intubation scenarios.

**Table 4:** Comparison of accuracy with the state-of-the-art methods on the clinical dataset.

Methods	Backbone	Detection accuracy (%)			Segmentation accuracy (%)		
		*mAP*^*box*^	${\boldsymbol{A}}{{\boldsymbol{P}}}_{{\bf{50}}}^{{\boldsymbol{b}}{\boldsymbol{o}}{\boldsymbol{x}}}$	${\boldsymbol{A}}{{\boldsymbol{P}}}_{{\bf{75}}}^{{\boldsymbol{b}}{\boldsymbol{o}}{\boldsymbol{x}}}$	*mAP*^*mask*^	${\boldsymbol{A}}{{\boldsymbol{P}}}_{{\bf{50}}}^{{\boldsymbol{b}}{\boldsymbol{o}}{\boldsymbol{x}}}$	${\boldsymbol{A}}{{\boldsymbol{P}}}_{{\bf{75}}}^{{\boldsymbol{b}}{\boldsymbol{o}}{\boldsymbol{x}}}$
ATSS^44^	ResNet50	17.3	37.1	13.8	–	–	–
BoxInst^45^	ResNet50	7.1	13.9	6.5	3.3	10.5	0.7
CondInst^46^	ResNet50	15.5	25.2	16.3	13.2	23.7	13.4
CMask RCNN^47^	ResNet50	25.4	47.4	24.0	24.2	46.3	21.3
Conditional DETR^48^	ResNet50	10.4	33.4	2.6	–	–	–
DAB DETR^49^	ResNet50	18.3	43.5	13.5	–	–	–
Deform. DETR^50^	ResNet50	28.7	58.9	27.3	–	–	–
DETR^51^	ResNet50	9.2	21.6	6.4	–	–	–
GFL^40^	ResNet101	33.7	58.4	35.0	–	–	–
Mask2Former^52^	ResNet50	16.2	27.6	17.4	14.9	27.1	14.5
Mask RCNN^53^	ResNet50	17.4	40.3	13.2	21.5	42.3	18.2
Mask RCNN^53^	Swin-T	18.1	39.0	14.1	20.6	39.1	17.8
MS RCNN^54^	ResNet50	19.0	42.4	13.7	23.2	43.9	20.6
PointRend^41^	ResNet50	23.8	50.9	19.1	25.5	51.4	19.9
QueryInst^55^	ResNet50	6.1	13.2	4.7	6.8	13.7	5.7
SOLOv2^56^	ResNet50	–	–	–	16.6	33.8	14.4
SparseInst^57^	ResNet50	–	–	–	14.7	31.7	13.0
RTMDet-Ins-s^39^	CSPNeXt	23.2	38.9	23.9	22.7	38.3	22.8
YOLACT^58^	ResNet50	14.1	35.5	8.3	16.4	33.8	13.4

## Data Availability

The dataset produced in this work has been deposited in the Figshare repository and is publicly available at 10.6084/m9.figshare.26342779.v4^38^.

## Code availability

The source code for data processing, model training, and evaluation is publicly available at: https://github.com/HBUT-CV/ScientificData. Our implementation is built upon the MMDetection framework^42^. To facilitate reproduction of the SOTA experimental results, users can select and run corresponding model configuration files located in the config directory of the repository. Detailed installation and execution instructions are provided in the repository’s documentation.
